# circRNAs and their relationship with breast cancer: a review

**DOI:** 10.1186/s12957-022-02842-5

**Published:** 2022-11-28

**Authors:** Fan Zhang, Liying Li, Zhimin Fan

**Affiliations:** Department of Breast Surgery, General Surgery Center, The First Hospital of Jilin University, Changchun, Jilin 130021 China

**Keywords:** circRNAs, Breast cancer, ceRNA, Clinic

## Abstract

**Background:**

Recently, an increasing number of studies have been conducted on circular RNAs (circRNAs) that have demonstrated their different roles in a variety of biological processes. Moreover, a large number of circRNAs have been shown to be involved in the occurrence and development of breast cancer (BC).

**Main body:**

Both functional and mechanistic experiments have shown that circular RNAs (circRNAs) can act as competing endogenous RNAs by sponging miRNAs, encoding proteins, and regulating parental genes. In doing so, circRNAs modulate the proliferation, migration, apoptosis, and invasion of BC cells in vitro as well as tumor growth and metastasis in vivo. Moreover, scores of circRNAs have been demonstrated to be related to clinicopathological features, prognosis, and treatment sensitivity in patients with BC; many circRNAs have shown potential as biomarkers for diagnosis, drug sensitivity, and prognosis prediction. Furthermore, researchers have focused on circRNAs as potential therapeutic targets.

**Conclusion:**

In this review, we briefly summarize the functions and categories of circRNAs, their different roles in BC, and recent research and therapeutic progress related to circRNAs.

## Background

Cancer remains a serious global problem. According to Global Cancer Statistics for the year 2020 [1], breast cancer (BC) was the most diagnosed cancer and caused the most cancer-related deaths among women. Furthermore, BC is estimated to account for almost one-third of all new cancer diagnoses and has the second highest mortality rate among women in the USA in 2022 [2].

Circular RNAs (circRNAs), characterized by a covalently closed loop structure without a 5′-cap or a 3′-polyadenylated tail, were first reported as viroids in 1976 [3] and are thought to be an error product of RNA splicing [4]. With the development of high-throughput RNA-sequencing technology and bioinformatics tools in recent years, an increasing number of circRNAs have been discovered and researched. Many circRNAs play important roles in physiological and pathological processes and are characterized by their variety, stable structure, conserved sequences, and specific expression in cells or tissues. In recent years, many studies have shown that circRNAs, as oncogenes or tumor suppressor genes, are closely related to the occurrence of various tumors such as BC [5, 6], endometrial cancer [7], cervical cancer [8], lung carcinoma [9], hepatocellular carcinoma [10], esophageal squamous cell carcinoma [11], and colorectal cancer [12].

CircRNAs are not only involved in regulating proliferation, invasion, and migration of BC cells but may serve as novel molecular markers for early diagnosis and individualized prognosis evaluation of BC. At present, reports on circRNAs related to BC are gradually increasing, but there are few summary articles on these RNAs. Clinical research is still in the early stages, and there remains a lack of comprehensive understanding. This study reviews the relationship between circRNAs and BC to provide new ideas for seeking more valuable circRNA-based diagnosis and personalized targeted therapy strategies.

## Biogenesis and classification of circRNAs

It is generally believed that the majority of circRNAs are generated by back-splicing or other processes of precursor messenger RNAs (pre-mRNAs) and are further processed by enzymes. In 2013, Jeck et al. [13] proposed two models of circRNA generation namely lariat-driven circularization and intron-pairing-driven circularization, which laid the foundation for further exploration of circRNA formation. The former is based on exon-skipping, which results in lariat structure and promotes circularization; the latter is based on Alu complementarity, and other RNA pairings of reverse complement, or repetitive sequences, which allow for circularization [13]. In addition to cis-acting elements (such as Alu-repeats), RNA-binding proteins (RBPs) like muscleblind-like protein 1 (MBNL1) [14], have also been shown to drive circRNA circularization. The canonical spliceosomal machinery can catalyze the process of back-splicing, which is modulated by both intronic complementary sequences and RBPs [15].

**Fig. 1:**
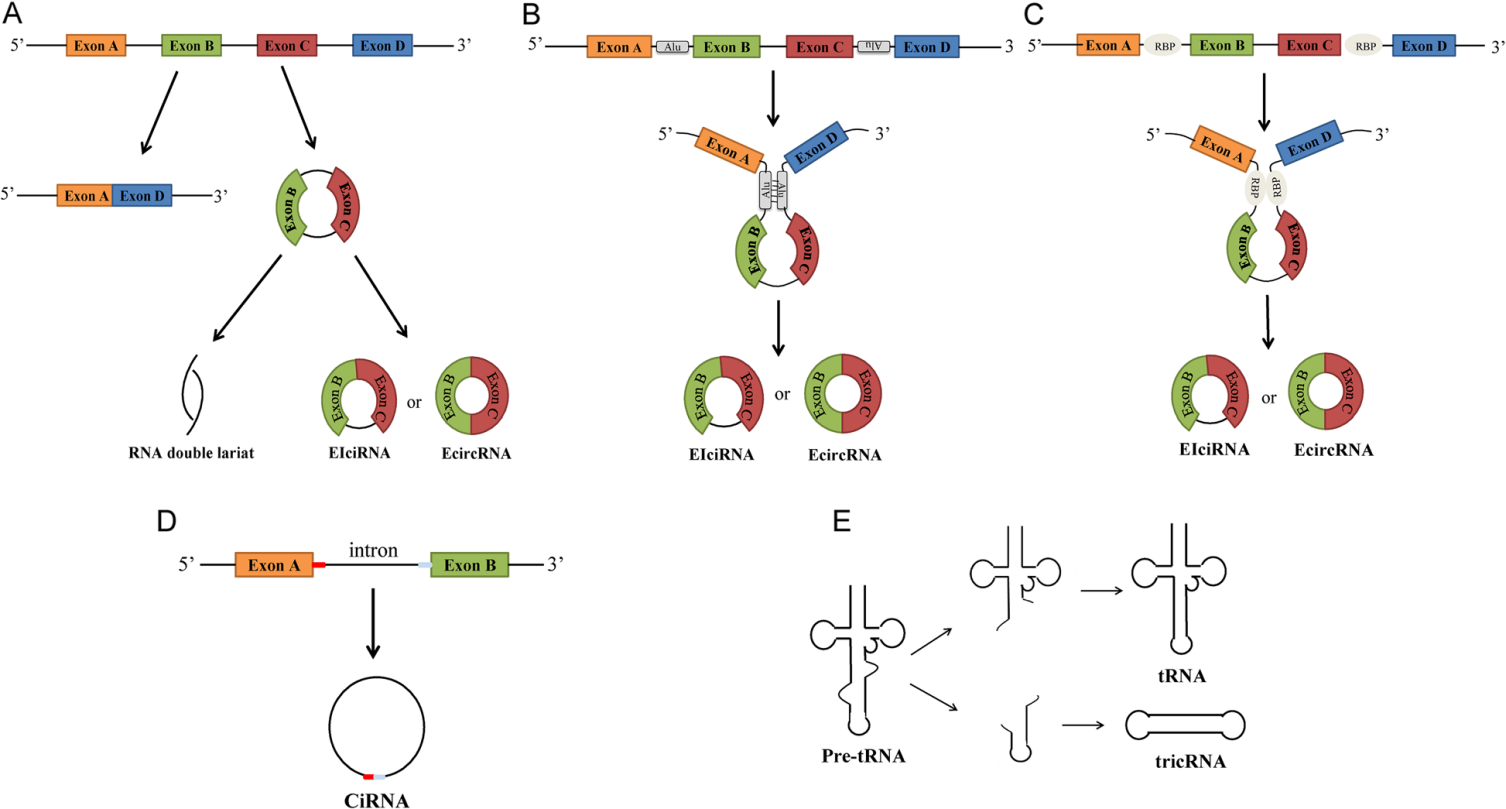
Biogenesis and classification of circRNAs. **A** Lariat-driven circularization. The 5' end of exon D combines with the 3' end of exon A to form a lariat structure. Then, one or both introns of the lariat structure are excised, resulting in the formation of EIciRNA or EcircRNA, respectively. **B**, **C** Intron-pairing-driven circularization. The Alu complementarity (**B**) or RBPs (**C**) located on the introns bring one another closer and thus promote the formation of EIciRNA or EcircRNA. **D** The 3′ and 5′ ends of the intron between exon A and exon B bind close to one another and cyclize, resulting in the formation of ciRNA. **E** Pre-tRNA is cut by the tRNA splicing endonuclease complex (TSEN) and the ends are connected to form tRNA and tricRNA

circRNAs can be divided into four main categories: (1) exonic circRNAs (EcircRNAs) are composed of one or more exons, are mainly located in the cytoplasm, and account for over 80% of the known circRNAs [16]; (2) circular intronic RNAs (ciRNAs) are comprised of introns and are mainly located in the nucleus; (3) exonic-intronic circRNAs (EIciRNAs) are composed of both exons and introns, and are also mainly located in the nucleus; (4) other circRNAs are generated by cyclization of the viral RNA genome [17, 18] or during the splicing of pre-tRNA [19] (tRNA intronic circRNAs, or tricRNAs). Figure 1 illustrates the biogenesis and classification of circRNAs.

## Properties of circRNAs

Compared to linear RNAs, circRNAs have been shown to be more abundant in eukaryotes. Studies have shown that EcircRNAs appear to be derived from over 14% of transcribed genes in human fibroblasts, and there are more than 25,000 different circRNAs that are expressed at much higher levels than their corresponding canonical mRNA [13, 20]. In some cases, circRNAs can be expressed 10 times more than their associated linear transcripts [13].

circRNAs have been shown to be more stable than the associated linear mRNAs [13]. Because of their covalently closed loop structure, which lacks a 5′-cap and 3′-polyadenylated tail, circRNAs are resistant to the activity of RNases, debranching enzymes, and RNA exonucleases. As a result, the average half-life of mRNAs in cells (10 h) is much shorter than that of circRNAs (> 48 h) [21]. However, circRNAs can also be degraded by some types of RNase R [22] and are regulated by siRNAs, shRNAs, and miRNAs.

Most circRNAs exhibit tissue-, cell-, and developmental stage-specificity [23, 24]. CiRS-7 has been reported to be overexpressed in brain tissues, compared to non-neuronal tissues with low to no expression [25]. Sequencing of RNA from several organs revealed lower numbers and expression levels of circRNAs in human adult tissues compared to fetal tissues, and 50% of circRNAs showed tissue-specificity expression patterns [26]. Most circRNAs are located in the nucleus, whereas most EcircRNAs are transported to the cytoplasm.

Sequence conservation is another characteristic of the circRNAs. Wang et al. found evidence for circRNAs throughout the eukaryotic tree of life which showed structural features similar to eukaryotes that diverged more than 1 billion years ago [27]. Rybak et al. analyzed the conservation of neuronal circRNAs between mammals using RNase R treatment and Sanger sequencing, which showed that the head-to-tail junction sequences were precisely conserved between humans and mice [28].

circRNAs are mainly released through exosomes, which are primarily derived from multivesicular bodies formed by the invagination of intracellular lysosomal particles. These are released into the extracellular matrix after the fusion of the outer membrane of vesicles with the cell membrane. Exosomes can be shed by most cell types and circulate in bodily fluids such as blood, saliva, breast milk, and urine [29, 30]. After being transported from the nucleus to the cytoplasm, circRNAs are secreted into bodily fluids by the exosomes. It has been reported that circRNAs can be transferred to recipient cells via exosome delivery, indicating that exosomal circRNAs are important for invasion and metastasis [31]. Based on this information, circRNAs are considered potential non-invasive biomarkers for the diagnosis and prognosis of various diseases.

## Functional mechanisms of circRNAs and the molecular mechanisms in BC

The biological function of circRNA has been widely studied, and the functional mechanism can be mainly divided into the following three types: miRNA sponging, protein/peptide template, and regulating parental genes. An increasing number of circRNAs have been found to be associated with BC tumorigenesis, progression, and metastasis. Furthermore, various circRNAs have been reported to be abnormally expressed in BC tissues and cell lines. The circRNAs that have been reported to be associated with BC are summarized and listed in Table 1, and their functions and mechanisms in BC are also demonstrated. In Table 2, the results of RNA-seq or microarray analysis of circRNAs associated with BC that have been reported in the literature are listed, including the total number of circRNAs, number of differentially expressed circRNAs, and cut-off criteria (fold change and *P* value) defining differential expression.

**Table 1:** circRNAs associated with breast cancer

circRNA	circRNA ID	Expression in BC	Function						Mechanism	Ref.
			Proliferation	Metastasis	Invasion	Migration	Mobility	Others		
hsa_circ_001783	hsa_circ_001783	↑	✓	✓					Sponging miR-200c-3p	[5]
circANKS1B	hsa_circ_0007294	↑		✓	✓	✓			Sponging miR-148a-3p and miR-152-3p	[32]
circAGFG1	-	↑	✓		✓		✓		Sponging miR-195-5p	[33]
circRNF20	hsa_circ_0087784	↑	✓					Warburg effect	Sponging miR-487a	[6]
hsa_circ_0005273	hsa_circ_0005273	↑	✓						Sponging miR-200a-3p	[34]
circFOXK2	hsa_circ_0000816	↑			✓	✓			Sponging miR-370	[35]
circRHOT1	-	↑	✓		✓	✓			Sponging miR-106a-5p	[36]
circRNA_103809	circRNA_103809	↓	✓		✓	✓			Sponging miR-532-3p	[37]
circSMARCA5	-	↓						Cisplatin response	Interacting with host gene	[38]
hsa_circ_0025202	hsa_circ_0025202	↓	✓			✓		Apoptosis, Tamoxifen Sensitivity	Sponging miR-182-5p	[39]
circTADA2A-E6	hsa_circ_0006220	↓	✓		✓	✓		Clonogenicity	Sponging miR-203a-3p	[40]
circNR3C2	hsa_circ_0071127	↓	✓		✓	✓			Sponging miR-513a-3p	[41]

**Table 2:** Overview of circRNAs identified by RNA sequencing and microarrays in BC

Sample	Subtype of BC	Detection method	Number of circRNAs	Number of differently expressed circRNAs (upregulated/ downregulated)	Fold Change	*P* value	Ref
4 pairs of BC and BCN tissues	TNBC	RNA-seq	–	354 (47/307)	≥ 2	< 0.05	[42]
3 pairs of BC and BCN tissues	TNBC	RNA-seq	69815	5033 (1307/3726)	≥ 2	< 0.05	[32]
4 pairs of BC and BCN tissues	IDC	circRNA microarray	–	1155 (715/440)	≥ 2	< 0.05	[43]
MCF7/TR and MCF7/P cells	HR-positive BC	RNA-seq	36110	465 (352/113)	> 1	< 0.01	[39]
three TNBC cell lines (MDA-MB-231, MDA-MB468 and BT549) and one HME cell line (MCF-10A)	TNBC	circRNA microarray	–	270 (93/174)	> 3	< 0.05	[44]
4 pairs of BC and para-cancer tissues	non-TNBC	circRNA microarray	–	85 (63 /22)	≥ 2	< 0.05	[45]
5 pairs of BC and paired normal tissues	TNBC	RNA-seq	27346	1200 (613/587)	≥ 2	< 0.01	[46]
5 pairs of plasma of BC patients and healthy controls	–	circRNA microarray	–	41 (19/22)	≥ 2	< 0.05	[43]
4 pairs of BC and para-cancer tissues	TNBC	circRNA microarray	–	250 (173/77)	≥ 1.5	< 0.05	[47]
BC and NMGT tissues	TNBC (4 BC, 3 NMGT)	circRNA microarray	–	215 (122/93)	–	–	[40]
	Luminal A (4BC, 3 NMGT)	circRNA microarray	–	73 (55/18)			

*NMGT* normal mammary gland tissues, *BCN tissues* normal breast tissues of patients with breast cancer

### miRNA sponging

miRNAs can base pair directly with target sites within the untranslated regions of mRNAs, and thus play key roles in regulating gene expression at the post-transcriptional level [48]. circRNAs contain miRNA binding sites. Thus, by competitively binding to miRNAs and acting as sponges, circRNAs can degrade mRNAs in a targeted manner and indirectly regulate gene expression, which may have an impact on tumor development and drug resistance [49]. This is the most important mechanism by which circRNAs play the role of regulation.

CiRS-7 was the first circRNA to be validated as a miRNA sponge and was verified to contain more than 70 selectively conserved binding sites for miR-7 [48]. Hsa_circRNA_101237 was found to be an important onco-circRNA of non-small cell lung cancer (NSCLC) that sponges miRNA-490-3p and thus promotes the expression of MAPK1 to drive proliferation, migration, and invasion of NSCLC cell lines [50].

A study on circRNA_0025202 found that its tumor inhibition and tamoxifen sensitization effects on HR-positive BC were achieved through sponging of miR-182-5p, as well as through further regulation of the expression and activity of its target protein FOXO3a [39]. Yang et al. reported that circAGFG1 may act as a competing endogenous RNA (ceRNA) by sponging miR-195-5p and regulating the expression of its target CCNE1, thus promoting triple-negative breast cancer (TNBC) progression [33]. circWAC has also been reported to affect the chemosensitivity of TNBC by sponging miR-142 and regulating its target WWP1 [51]. A study on circANKS1B showed that it was associated with TNBC metastasis, advanced stage, and overall survival (OS) as a result of sponging miR-148a-3p and miR-152-3p [32]. Cao et al. reported that circRNG20 promoted BC proliferation via the circRNF20/miR-487a/HIF-1α/HK2 axis [6]. circFOXK2 and circRNA_103809 have also been reported to promote the invasion and migration of BC by sponging miR-370 and miR-532-3p, respectively [35, 37]. Xu et al. reported that circTADA2A-E6 acts as a miR-203a-3p sponge and inhibits the expression of SOCS3, the target gene of miR-203a-3p, thus suppressing cell proliferation, migration, invasion, and clonogenicity in vitro [40]. A study on circCD44 (hsa_circ_0021735), a type of EcircRNA that is mostly localized in the cytoplasm, showed that it was associated with TNBC proliferation and malignant characteristics as well as prognosis [52]. Furthermore, circCD44 was predicted to act as a ceRNA and to mediate KRAS degradation through targeting of miR-502-5p [52].

circERPT9, a novel circRNA generated from exon 2 of SERPT9 by back-splicing, is involved in the carcinogenesis and development of TNBC [42]. Functional experiments demonstrated that circERPT9 promotes BC cell proliferation, migration, and invasion while suppressing cell cycle arrest, apoptosis, and autophagy. Further bioinformatic analysis showed that circSEPT9 can sponge miR-637 and activate the LIF-STAT3 pathway through the circSEPT9/miR-637/LIF axis in TNBC. Furthermore, E2F1 and EIF4A3 could promote the expression of circSEPT9, although the mechanism involved remains unclear.

Hsa_circ_001783 has a genomic sequence 34460 nucleotides (nt) in length and is a type of EIciRNA [5]. Functional experiments showed that transfection of siRNAs targeting the junction site of hsa_circ_001783 inhibited the progression of BC cells. Further results showed that hsa_circ_001783 could sponge miR-200c-3p and upregulate its targeting genes.

In the analysis of a large cohort, hsa_circ_0005273 was substantially expressed in BC tissues. This circRNA is 357 base pairs in length and is derived from the circularization of exons 27–29 of the PTK2 gene. According to functional experiments, by sponging miR-200a-3p, hsa_circ_0005273 can boost BC cell proliferation both in vitro and in vivo, thereby regulating the miR-200a-3p/YAP1 axis and inactivating the Hippo signaling pathway [34].

circNFIC (hsa_circ_0002018) has the potential to be a new suppressive factor and BC biomarker [53]. Overexpression of circNFIC suppresses proliferation and migration of BC cells both in vitro and in vivo. Both bioinformatic analysis and a series of experiments demonstrated that circNFIC could sponge miR-658 and restore the expression of its target gene, UPK1A. Additionally, further assays revealed that circNFIC overexpression increased the enrichment of circNFIC on Ago2 but decreased that of UPK1A. However, UPK1A expression could be promoted, indicating that circNFIC may act as a ceRNA through the circNFIC/miR-658/UPK1 axis. In summary, circNFIC was demonstrated to be a novel anti-oncogenic circRNA that regulates BC proliferation and migration through the circNFIC/miR-658/UPK1 regulatory network.

circ_0000442 (hsa_circ_0000442), derived from the MED13L gene, is downregulated in BC cells and tissues compared to that in adjacent non-cancerous cells [54]. In a subsequent functional study, results showed that circ_0000442 suppressed the proliferation of BC both in vivo and in vitro and thus acted as a tumor suppressor. Furthermore, circ_0000442 functions as a sponge for miR-148b-3p, which promotes cell proliferation. Further exploration of the underlying mechanism showed that PTEN was the direct target gene of miR-148b-3p, which was identified as a negative PI3K/Akt pathway switch. Overexpression of circ_0000442 increased the level of PTEN while suppressing phosphorylation of PI3K and Akt; however, overexpression of miR-148b-3p reversed this phenomenon. Taken together, circ_0000442 may play a role in inhibiting BC tumor progression through the circ_0000442/miR-148b-3p/PTEN/PI3K/Akt axis.

Recently, the concept of autophagy has been proposed to promote the progression of BC [55, 56]. Autophagy is an important mechanism for the survival of disseminated dormant BC cells and plays a key role in tumor metastasis and recurrence [55]. circCDYL was shown to have up to 3.2-fold higher expression in BC tissues than that in adjacent non-cancerous tissues, and was associated with poor prognosis and clinical response in patients with BC [57]. Overexpression or silencing of circCDYL is related to the level of LC3-II protein, which is an autophagic marker. Biological function experiments showed that circCDYL regulated the proliferation of BC cells through the miR-1275-ATG7/ULK1-autophagic axis.

circANKS1B, arising from exons 5 to 8 of the ANKS1B gene, is markedly overexpressed in TNBC compared to normal tissues and other subtypes of BC [32]. Functional experiments have shown that circANKS1B promotes the epithelial-mesenchymal transition (EMT) to facilitate BC invasion and metastasis both in vitro and in vivo, but has no effect on BC growth. The level of the epithelial cell marker E-cadherin was increased, while the level of mesenchymal cell markers vimentin and fibronectin were decreased. Further mechanistic studies revealed that the ESRP1/circANKS1B/miR-148a/152-3p/USF1 axis regulates BC via TGF-β1-mediated EMT.

circNR3C2, which is generated by back-splicing of the second exon of the nuclear receptor subfamily 3 group C member 2 (NR3C2) located at chr4:149356255–149358014 (1760 nt), was markedly under expressed in TNBC, and a negative correlation was found in clinical data [41]. HMG-CoA reductase degradation protein 1 (HRD1) was also found to be under expressed in TNBC and was negatively correlated with vimentin expression. Functional experiments showed that overexpression of HRD1 suppressed proliferation, migration, and invasion capabilities of BC cells, and these effects were reversed upon overexpression of vimentin. Further mechanistic studies suggested that circNR3C2 regulates the tumor-suppressive effect of HRD1 by sponging miR-513a-3p in TNBC.

Ferroptosis, a newly discovered form of programmed cell death characterized by the iron-dependent accumulation of lipid reactive oxygen species (ROS), plays an important role in BC and other diseases. Intracellular lipid ROS are catalyzed by Fe^2+^-containing enzymes, and excessive ROS are removed through the antioxidant system. Cell death occurs when the excess accumulation of ROS caused by lipid peroxidation exceeds the safety threshold [58–60]. circRHOT1 has been shown to promote proliferation, invasion, and migration of BC cells both in vivo and in vitro while inhibiting apoptosis and ferroptosis. Furthermore, overexpression of STAT3 and the use of a miR-106a-5p inhibitor reversed BC progression that was mediated by circRHOT1 knockdown and also increased the levels of ROS, iron, and Fe^2+^. Taken together, this study suggests that circRHOT1 promotes BC progression via the miR-106a-5p/STAT3 axis [36].

### Protein/peptide template

circRNAs were originally considered noncoding RNAs because of their special structure, which does not provide suitable ribosomal entry sites. However, it was first reported in 1995 that circRNAs with internal ribosome entry site (IRES) elements could be translated by eukaryotic ribosomes [61]. Recently, an increasing number of studies have demonstrated that a special group of circRNAs can encode proteins [62–64].

circ-ZNF609 was found to contain an open reading frame (ORF) spanning the start codon that could be translated into protein in a splicing-dependent and cap-independent manner [62]. Another study showed that circ-SHPRH contains an ORF driven by an IRES. Thus, circ-SHPRH can be translated into a novel protein, shprh-146aa, which is a tumor suppressor in human glioblastoma [63]. Another recent study showed that circDIDO1, a circRNA containing IRES, ORF, and m6A modification, could generate a 529 amino acid tumor suppressor protein that inhibits the activity of poly ADP-ribose polymerase 1 through direct interaction [65].

### Regulating parental genes

**Fig. 2:**
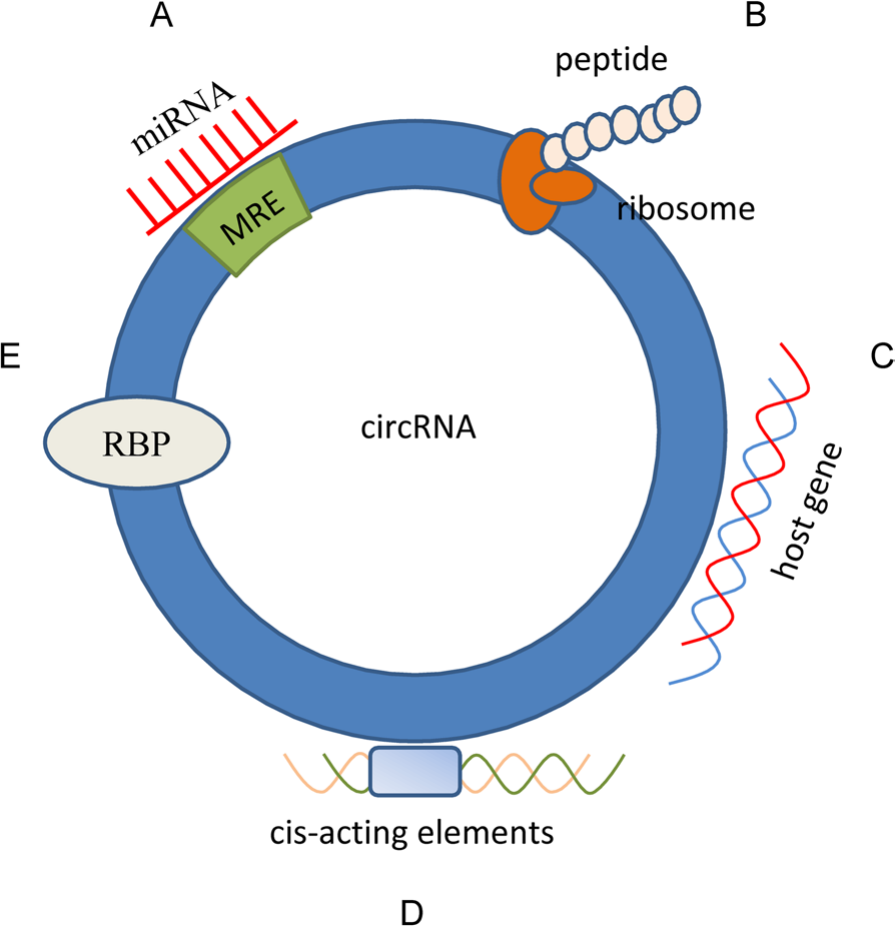
Functional mechanisms of circRNAs. **A** miRNA sponging. circRNAs are rich in miRNA binding sites (miRNA response elements, MRE) and act as miRNA sponges to prevent miRNA from interacting with mRNA, thus indirectly regulating the expression of downstream target genes of miRNA. **B** Protein/peptide template. Some circRNAs can be translated by ribosomes and encode peptides to perform regulatory functions. **C–E** Regulating parental genes. Some circRNAs can bind with host genes (**C**) or cis-acting elements (**D**), thus directly or indirectly modulating host gene transcription. In addition, circRNAs can change splicing patterns or mRNA stability by binding to RNA-binding proteins (RBPs) (**E**), thus regulating parental gene transcription

Another important function of circRNAs is the regulation of parental gene expression. First, circRNAs can directly interact with host genes. A study showed that the circRNA generated from exon 6 of the SEPALLATA3 gene in plants binds to its cognate DNA locus to form RNA: DNA hybrids or R-loops, resulting in transcriptional pausing; whereas, cognate linear RNA binds less strongly to DNA [66]. This was the first time that circRNA manipulation was found to mediate phenotypes at the organismal level [66]. It was recently reported that circSMARCA5 inhibits the expression of SMARC5 by forming R-loops with its parent gene. This results in a transcriptional halt at exon 15 of SMARCA5, thereby inhibiting its DNA damage repair function in cancer cells [38]. Second, circRNAs can combine with cis-acting elements (e.g., promoters) directly or indirectly to modulate host gene transcription. A previous study illustrated that circTulp4, which is mainly located in the nucleus, regulates the function of the nervous system and is likely involved in the development of Alzheimer’s disease. This regulation occurs via interactions with U1 small nuclear ribonucleoprotein particles and RNA polymerase II at the promoter to regulate the transcription of its parental gene [67]. Third, circRNAs can bind to RBPs to regulate transcription. RBPs play key roles in regulating RNA at the post-transcriptional level and are involved in tissue development and disorders [68]. By interacting with specific cis-regulatory elements, RBPs assemble the ribonucleoprotein complex to bind RNA sequences and influence the expression and function of their target RNAs [69]. A previous study proposed that the connection between HuR and PABPN1 mRNA could be prevented, and the translation of PABPN1 could be reduced due to the wide binding of circPABPN1 to HuR. This provided the first concrete example of translation affected by the competition between a circRNA and its cognate mRNA to bind to RBP [70]. Similarly, cia-cGAS inhibits cGAS gene expression by binding to the cGAS protein, thereby further inhibiting the generation of type I interferon and ultimately dormant hematopoietic stem cells in the bone marrow [71]. Figure 2 shows the main functional mechanisms of circRNAs.

## Correlation between circRNA expression and clinicopathological features of BC

circRNAs can promote or suppress BC progression by sponging miRNAs, encoding proteins, and regulating parental genes. These effects are reflected in the modulation of proliferation, migration, and invasion in vitro and in vivo through regulation of tumor growth, metastasis, and other clinicopathological features. The reported circRNAs associated with clinicopathological features of BC are listed in Table 3. Additionally, the number of cases, subtypes of BC, and related clinicopathological features are also demonstrated.

**Table 3:** circRNAs and clinicopathological features of breast cancer

CircRNA	Expression in BC	Subtype of BC	Clinicopathological features					Number of cases	Ref.
			Tumor size and TNM stage	Metastasis	IHC and subtype	Grade	Others		
circSEPT9	↑	TNBC	T, N, TNM					60	[42]
hsa_circ_001783	↑	-	T, N, TNM		ER, PR, Ki-67, molecular subtype			136	[5]
circRNF20 (hsa_circ_0087784)	↑	-	tumor size	LN				50	[6]
hsa_circ_0005273	↑	–	tumor size, TNM	LN, distant				120	[34]
circACTN4	↑	Non-TNBC	T, N, TNM					240	[45]
hsa_circ_0001785	↑	–	TNM	Distant		Histological grade		57	[43]
circHMCU (hsa_circ_0000247)	↑	–	T, N	LN	ER, PR, HER2	Histological grade		267	[72]
circKIF4A (hsa_circ_0007255)	↑	TNBC	Tumor size, TNM	LN				240	[73]
circMMP11 (hsa_circ_0062558)	↑	–	TNM	LN				113	[74]
circEPSTI1 (hsa_circ_000479)	↑	TNBC	Tumor size, TNM	LN				240	[75]
circLARP4	↑	–	Tumor size, T, N, TNM					283	[76]
circPLK1 (has_circ_0038632)	↑	TNBC	Tumor size, TNM	LN				240	[75]
circRNA_100395	↓	–	T					52	[77]
hsa_circ_0025202	↓	HR-positive BC		LN		Histological grade,		230	[39]
hsa_circ_0001073	↓	-	Tumor size	LN			age	132	[78]

*LN* lymph node, *IHC* immunohistochemistry, *ER* estrogen receptor, *PR* progesterone receptor, *HER2* human epidermal growth factor receptor 2

A study conducted on a cohort of 120 patients with BC showed that hsa_circ_0005273 expression was positively associated with lymph node metastasis, tumor size, TNM stage, and distant metastasis but was not correlated with age [34]. A comparison of 60 TNBC samples and adjacent non-cancerous tissues showed that circSEPT9 was positively correlated with T, N, and TNM stages [42]. A cohort of 240 BC tissues demonstrated that circACTN4 expression was positively correlated with T, N, and TNM stages [45]. A study showed that circAGFG1 expression level is associated with poor prognosis as well as the T and N stages of the TNM staging system [33]. circNFIC was also reported to be downregulated in BC, and negatively correlated with lymph node metastasis (*P* = 0.021) as well as poor prognosis (*P* = 0.002) [53].

## CircRNAs as diagnostic and prognostic biomarkers for BC

Early diagnosis and prognosis are two of the most important issues for patients with BC, and circRNAs show great potential as biomarkers. Clinical diagnostic and prognostic values of circRNAs have been explored in several studies. The reported circRNAs with diagnostic and prognostic potential and their related indicators are listed in Tables 4 and 5, demonstrating that circRNA has great potential for the diagnosis and prognosis of BC.

**Table 4:** Diagnosis efficiency of circRNAs for breast cancer

CircRNA	Expression in BC	Subtype of BC	Sample	AUC	Sensitivity	Specificity	Cut-off	Number of cases	Ref.
circACTN4	↑	non-TNBC	Tissue	0.719	0.7	0.7	12.77	80	[45]
hsa_circ_0001785	↑	–	Plasma	0.771	0.786	0.756	–	20	[43]
				0.784	0.764	0.699	0.981	57	
circSEPT9	↑	TNBC	Tissue	0.711	0.633	0.75	1.971	60	[42]
hsa_circ_103110	↑	IDC	Tissue	0.63	0.63	0.63	8.97	51	[79]
hsa_circ_104689				0.61	0.57	0.55	7.67		
hsa_circ_104821				0.60	0.57	0.57	6.04		
hsa_circ_006054	↓			0.71	0.65	0.69	14.84		
hsa_circ_100219				0.78	0.69	0.71	8.95		
hsa_circ_406697				0.64	0.63	0.63	14.24		
hsa_circ_0001073	↓	–	Tissue	0.9898	0.9242	0.9737	6.68	132	[78]
circVRK1	↓	–	Tissue	0.72	0.617	0.791	0.425	350	[80]

*IDC* invasive ductal carcinoma

**Table 5:** circRNAs and prognosis of breast cancer

CircRNA	Expression in BC	Subtype of BC	Sample	Survival	*P* value	HR (95%CI)	Number of cases	Ref.
circANKS1B	↑	–	Tissue	OS	0.002	3.29 (1.75–8.23)	165	[32]
circCDYL	↑	–	Tissue	DFS	0.0036	2.85 (1.415–5.739)	113	[57]
		MBC	Serum	OS	0.002	3.748 (1.233–11.239)	30	
circAGFG1	↑	TNBC	Tissue	OS	< 0.0001	–	80	[33]
circKIF4A	↑	TNBC	Tissue	DFS, OS	< 0.01	–	240	[73]
circFBXW7	↑	TNBC	Tissue	OS	0.001	0.215 (0.119–0.387)	473	[81]
circ_0001073	↓	–	Tissue	RFS	0.038	–	132	[78]

*RFS* relapse-free survival, *DFS* disease-free survival, *OS* overall survival, *MBC* metastatic breast cancer

Receiver operating characteristic (ROC) curve analysis was used to assess the diagnostic value of circSEPT9 for TNBC screening. The area under the curve (AUC) of circSEPT9 was 0.711, and its specificity and sensitivity were 75.0% and 63.3%, respectively, with a cut-off value of 1.971; these values indicate that circSEPT9 may be an effective biomarker for TNBC diagnosis [42]. In a study conducted by Lü et al. [79], a circRNA microarray was used to identify differentially expressed circRNAs between BC and adjacent non-cancerous tissues. Validation experiments demonstrated upregulated expression of hsa_circ_103110, hsa_circ_104689, and hsa_circ_104821 in BC tissues, along with downregulated expression of hsa_circ_006054, hsa_circ_100219, and hsa_circ_406697. The AUC values were 0.63, 0.61, 0.60, 0.71, 0.78, and 0.64, respectively. In contrast, when hsa_circ_006054, hsa_circ_100219, and hsa_circ_406697 were used in combination, the AUC value increased to 0.82 (95% CI 0.73–0.90), providing strong evidence for circRNAs as diagnostic markers. Yin et al. [43] found that a novel circRNA, hsa_circ_0001785, was upregulated in the plasma of patients with BC via microarray analysis. ROC curve analysis of 20 paired plasma samples showed that the AUC value of has_circ_0001785 was 0.771 (95% CI 0.592–0.915). Another analysis of 57 paired plasma samples showed that the AUC values of CEA, CA-153, and hsa_circ_0001785 were 0.562, 0.639, and 0.784, respectively. The AUC value of the combination of the three potential biomarkers was 0.839, indicating their potential as biomarkers for BC.

Kaplan–Meier analysis performed on a large cohort suggested that BC patients with higher levels of hsa_circ_001783 were more likely to have poor disease-free survival (DFS) (*P* < 0.001). Multivariate analysis showed that the expression level of hsa_circ_001783 was an independent risk factor for BC prognosis (HR 9.114; 95% CI 2.428–34.206, *P* = 0.001) [5].

circTADA2A-E6, a circRNA spliced from exon 6 of TADA2A (chr17, 35766977–35839830), suppresses cell proliferation, migration, invasion, and clonogenicity in vitro. Combined with clinical data, downregulation of circTADA2A-E6 is associated with poor prognosis for TNBC [40].

circRGPD6 was dramatically reduced in BC tissues compared to healthy tissues [82]. Kaplan-Meier survival analysis suggests that BC patients with high expression of circRGPD6 or YAF2 are more likely to have superior DFS and OS than those with low expression, whereas miR-26b showed the opposite phenomenon. Further analysis and experiments revealed that transcript variant (TV) circRGPD6 suppresses BC stem cell-mediated metastasis through the miR-26b/YAF2 axis, further explaining these results. These findings further demonstrate that many circRNAs show great value in the prognosis of BC.

## circRNAs expression and treatment of BC

### circRNAs predict drug sensitivity for BC

Given their high stability, abundance, and sequence conservation, circRNAs are considered to be potential biomarkers of drug sensitivity. For instance, a study of hsa_circ_0025202 demonstrated that overexpression of hsa_circ_0025202 sensitized cells to tamoxifen treatment [39].

TNBC tissues lack expression of estrogen receptor (ER), progesterone receptor (PR), and human epidermal growth factor receptor 2 (HER2). Therefore, we believe that anti-HER2-targeted therapy is not beneficial for patients with TNBC. However, Lee et al. found that circ-HER2 is enriched in TNBC, which in turn encodes HER2-103. In vitro experiments showed that HER2-103 promotes proliferation, invasion, and metastasis of TNBC cells. HER2-103 binds to EGFR and/or HER3 and activates the PI3K-Akt pathway, thereby promoting TNBC occurrence and progression. Pertuzumab has been shown to significantly reduce tumor growth and inhibit lung metastasis of TNBC cells with high HER2-103 expression in animal models [46].

Chemotherapeutic resistance is a critical aspect of TNBC treatment, and an effective biomarker for chemotherapeutic resistance prediction would play a key role in individualized treatment. circWAC is highly expressed and is negatively associated with prognosis in patients with TNBC [51]. Functional experimental data showed that circWAC had no obvious effect on metastasis and cell invasion but showed a strong relationship with chemosensitivity to paclitaxel. Subsequent studies have demonstrated that circWAC increases cisplatin resistance in BC cells, both in vivo and in vitro. Bioinformatic analysis and functional experiments further supported that circWAC can regulate chemotherapeutic resistance, specifically by sponging miR-142 and further targeting WWP1. Further analysis and experiments showed that miR-142 overexpression inhibited the PI3K/AKT signaling pathway. In summary, the circWAC/miR-142/WWP1 axis can affect TNBC chemosensitivity by regulating the PI3K/AKT signaling pathway.

### circRNAs as potential therapeutic targets for BC

As the regulatory roles of circRNAs are gradually being discovered, some may be investigated as effective therapeutic targets [20]. With increased biotechnological developments, many researchers have focused on circRNA-directed therapeutics [41, 45, 83]. For example, circDnmt1 is significantly upregulated in BC and promotes tumor growth and autophagy. Combining gold nanoparticles (PEG-AuNPs) with short interfering RNA (siRNA) targeting circDnmt1 was shown to inhibit these effects in mice [84]. circSka3 is highly expressed in patients with BC, and experiments have shown that it can promote tumor invasion and metastasis. In vivo experiments in nude mice confirmed that silencing circSka3 with siRNA could reverse these effects. Therefore, circSka3 may become a potential target in BC [85].

Currently, circRNA-targeted therapy research is in full swing, though it remains in the stage of preclinical animal model experiments [86]. Oncogenic circRNAs can be selectively inhibited or degraded using antisense RNA technology, such as RNA interference (RNAi) [87], CRISPR/Cas9 [88], CRISPR/Cas13 [89], and conditional circRNA knockout or knockdown [90]. Anti-oncogenic circRNAs can also be overexpressed using circRNA expression vectors [91], direct synthesis, and purification [92]. In addition, nanoparticle [93] and exosome [94] drug delivery systems are also being developed vigorously, providing a more solid foundation for the realization of circRNA-targeted therapy.

Researchers developed a versatile transcript variant (TV) amplifier delivery system, which targeted transgene expression in breast tumors with activity comparable to or stronger than the cytomegalovirus promoter in cancer cells [82, 95, 96]. A TV delivery system for microRNA (miR)-34a has also been developed, which reduced the proliferation of breast cancer stem cells (BCSCs) [23]. Furthermore, a new TV-circRGPD6 nanoparticle that selectively expresses circRGPD6 in metastatic BCSCs was generated recently [82] and inhibited both the tumor-promoting effect of BCSCs in vitro as well as the BC tumor metastasis-promoting effect in vivo. This nanoparticle also inhibited the proliferation and metastasis of BC via synergistic action with docetaxel. In conclusion, the development of TV-circRGPD6 nanoparticles may help reduce BC metastasis and provides a novel direction along with a potential tool for BC treatment.

## Conclusion and perspective

circRNAs are a group of novel RNAs that play key roles in tumorigenesis and progression of many types of benign and malignant tumors. Thus, research on their functions and mechanisms has increased and has provided more extensive information about circRNAs. Researchers have discovered that circRNAs can act as ceRNAs by sponging miRNAs, encoding proteins, and regulating parental genes. However, there are still many phenomena that cannot be explained by existing theories and hypotheses. Furthermore, circRNAs are being integrated into clinical data reporting. Thus, circRNAs are showing great potential as biomarkers for the diagnosis and prognosis of BC. Some circRNAs have also shown the potential to predict therapeutic outcomes and may become new therapeutic targets in the future. However, in general, research related to circRNA in tumors is still in its infancy, and the precise mechanisms involved have not been fully elucidated, which suggests that we still need to carry out many exploratory experiments. It is undeniable that circRNAs show great potential in tumor diagnosis, prognosis, and treatment, and they also have high clinical value. It is believed that with the unremitting efforts and practical exploration of scientists, circRNAs will open up a new field for tumor research, hopefully creating a new starting point for precision medicine in clinical diagnosis and treatment.

## Data Availability

All data generated or analyzed during this study are included in this published article.

## Abbreviations

circRNAs: Circular RNAs
BC: Breast cancer
ceRNA: Competing endogenous RNA
pre-mRNAs: Precursor messenger RNAs
RBPs: RNA-binding proteins
ICSs: Intronic complementary sequences
EcircRNAs: Exonic circRNAs
ciRNAs: Circular intronic RNAs
EIciRNAs: Exonic-intronic circRNAs
tricRNAs: tRNA intronic circRNAs
NSCLC: Non-small cell lung cancer
TNBC: Triple-negative breast cancer
ORF: Open reading frame
IRES: Internal ribosome entry site
RNP: Ribonucleoprotein
HR: Hazard ratio
FUBP1: Far upstream element binding protein 1
FUSE: Far upstream element
FIR: FBP interacting repressor
EMT: Epithelial-to-mesenchymal transition
NR3C2: Nuclear receptor subfamily 3 group C member 2
HRD1: HMG-CoA reductase degradation protein 1
ROS: Reactive oxygen species
AUC: Area under the curve
CI: Confidence interval
DFS: Disease-free survival
OS: Overall survival
ER: Estrogen receptor
PR: Progesterone receptor
HER2: Human epidermal growth factor receptor 2
TV: Transcript variant
miRNA: MicroRNA
NMGT: Normal mammary gland tissues
RFS: Relapse-free survival
BCSCs: Breast cancer stem cells

## References

[1] Sung H, Ferlay J, Siegel RL, Laversanne M, Soerjomataram I, Jemal A (2021). Global cancer statistics 2020: GLOBOCAN estimates of incidence and mortality worldwide for 36 cancers in 185 countries. CA Cancer J Clin.

[2] Siegel RL, Miller KD, Fuchs HE, Jemal A (2022). Cancer statistics, 2022. CA Cancer J Clin.

[3] Sanger HL, Klotz G, Riesner D, Gross HJ, Kleinschmidt AK (1976). Viroids are single-stranded covalently closed circular RNA molecules existing as highly base-paired rod-like structures. Proc Natl Acad Sci U S A.

[4] Cocquerelle C, Mascrez B, Hétuin D, Bailleul B (1993). Mis-splicing yields circular RNA molecules. FASEB J.

[5] Liu Z, Zhou Y, Liang G, Ling Y, Tan W, Tan L (2019). Circular RNA hsa_circ_001783 regulates breast cancer progression via sponging miR-200c-3p. Cell Death Dis.

[6] Cao L, Wang M, Dong Y, Xu B, Chen J, Ding Y (2020). Circular RNA circRNF20 promotes breast cancer tumorigenesis and Warburg effect through miR-487a/HIF-1α/HK2. Cell Death Dis.

[7] Liu Y, Chen S, Zong ZH, Guan X, Zhao Y (2020). CircRNA WHSC1 targets the miR-646/NPM1 pathway to promote the development of endometrial cancer. J Cell Mol Med.

[8] Chen RX, Liu HL, Yang LL, Kang FH, Xin LP, Huang LR (2019). Circular RNA circRNA_0000285 promotes cervical cancer development by regulating FUS. Eur Rev Med Pharmacol Sci.

[9] Wang J, Zhao X, Wang Y, Ren F, Sun D, Yan Y (2020). circRNA-002178 act as a ceRNA to promote PDL1/PD1 expression in lung adenocarcinoma. Cell Death Dis.

[10] Huang G, Liang M, Liu H, Huang J, Li P, Wang C (2020). CircRNA hsa_circRNA_104348 promotes hepatocellular carcinoma progression through modulating miR-187-3p/RTKN2 axis and activating Wnt/β-catenin pathway. Cell Death Dis.

[11] Liu J, Xue N, Guo Y, Niu K, Gao L, Zhang S (2019). CircRNA_100367 regulated the radiation sensitivity of esophageal squamous cell carcinomas through miR-217/Wnt3 pathway. Aging (Albany NY).

[12] Yang H, Li X, Meng Q, Sun H, Wu S, Hu W (2020). CircPTK2 (hsa_circ_0005273) as a novel therapeutic target for metastatic colorectal cancer. Mol Cancer.

[13] Jeck WR, Sorrentino JA, Wang K, Slevin MK, Burd CE, Liu J (2013). Circular RNAs are abundant, conserved, and associated with ALU repeats. Rna.

[14] Ashwal-Fluss R, Meyer M, Pamudurti NR, Ivanov A, Bartok O, Hanan M (2014). circRNA biogenesis competes with pre-mRNA splicing. Mol Cell.

[15] Li X, Yang L, Chen LL (2018). The biogenesis, functions, and challenges of circular RNAs. Mol Cell.

[16] Zaiou M (2019). Circular RNAs as potential biomarkers and therapeutic targets for metabolic diseases. Adv Exp Med Biol.

[17] Tagawa T, Gao S, Koparde VN, Gonzalez M, Spouge JL, Serquiña AP (2018). Discovery of Kaposi's sarcoma herpesvirus-encoded circular RNAs and a human antiviral circular RNA. Proc Natl Acad Sci U S A.

[18] Qiao Y, Zhao X, Liu J, Yang W (2019). Epstein-Barr virus circRNAome as host miRNA sponge regulates virus infection, cell cycle, and oncogenesis. Bioengineered.

[19] Schmidt CA, Giusto JD, Bao A, Hopper AK, Matera AG (2019). Molecular determinants of metazoan tricRNA biogenesis. Nucleic Acids Res.

[20] Li R, Jiang J, Shi H, Qian H, Zhang X, Xu W (2020). CircRNA: a rising star in gastric cancer. Cell Mol Life Sci.

[21] Li HM, Ma XL, Li HG (2019). Intriguing circles: conflicts and controversies in circular RNA research. Wiley Interdiscip Rev RNA.

[22] Vincent HA, Deutscher MP (2006). Substrate recognition and catalysis by the exoribonuclease RNase R. J Biol Chem.

[23] Kristensen LS, Andersen MS, Stagsted LVW, Ebbesen KK, Hansen TB, Kjems J (2019). The biogenesis, biology and characterization of circular RNAs. Nat Rev Genet.

[24] Huang A, Zheng H, Wu Z, Chen M, Huang Y (2020). Circular RNA-protein interactions: functions, mechanisms, and identification. Theranostics.

[25] Memczak S, Jens M, Elefsinioti A, Torti F, Krueger J, Rybak A (2013). Circular RNAs are a large class of animal RNAs with regulatory potency. Nature.

[26] Yang W, Li Y, Song X, Xu J, Xie J (2017). Genome-wide analysis of long noncoding RNA and mRNA co-expression profile in intrahepatic cholangiocarcinoma tissue by RNA sequencing. Oncotarget.

[27] Wang PL, Bao Y, Yee MC, Barrett SP, Hogan GJ, Olsen MN (2014). Circular RNA is expressed across the eukaryotic tree of life. PLoS One.

[28] Rybak-Wolf A, Stottmeister C, Glažar P, Jens M, Pino N, Giusti S (2015). Circular RNAs in the mammalian brain are highly abundant, conserved, and dynamically expressed. Mol Cell.

[29] Wang Y, Liu J, Ma J, Sun T, Zhou Q, Wang W (2019). Exosomal circRNAs: biogenesis, effect and application in human diseases. Mol Cancer.

[30] Boriachek K, Islam MN, Möller A, Salomon C, Nguyen NT, Hossain MSA, et al. Biological functions and current advances in isolation and detection strategies for exosome nanovesicles. Small. 2018:14. 10.1002/smll.201702153. 10.1002/smll.201702153 29282861

[31] Yan Y, Fu G, Ye Y, Ming L (2017). Exosomes participate in the carcinogenesis and the malignant behavior of gastric cancer. Scand J Gastroenterol.

[32] Zeng K, He B, Yang BB, Xu T, Chen X, Xu M (2018). The pro-metastasis effect of circANKS1B in breast cancer. Mol Cancer.

[33] Yang R, Xing L, Zheng X, Sun Y, Wang X, Chen J (2019). The circRNA circAGFG1 acts as a sponge of miR-195-5p to promote triple-negative breast cancer progression through regulating CCNE1 expression. Mol Cancer.

[34] Wang X, Ji C, Hu J, Deng X, Zheng W, Yu Y (2021). Hsa_circ_0005273 facilitates breast cancer tumorigenesis by regulating YAP1-hippo signaling pathway. J Exp Clin Cancer Res.

[35] Zhang W, Liu H, Jiang J, Yang Y, Wang W, Jia Z (2021). CircRNA circFOXK2 facilitates oncogenesis in breast cancer via IGF2BP3/miR-370 axis. Aging (Albany NY).

[36] Zhang H, Ge Z, Wang Z, Gao Y, Wang Y, Qu X (2021). Circular RNA RHOT1 promotes progression and inhibits ferroptosis via mir-106a-5p/STAT3 axis in breast cancer. Aging (Albany NY).

[37] Liu M, Luo C, Dong J, Guo J, Luo Q, Ye C (2020). CircRNA_103809 suppresses the proliferation and metastasis of breast cancer cells by sponging microRNA-532-3p (miR-532-3p). Front Genet.

[38] Xu X, Zhang J, Tian Y, Gao Y, Dong X, Chen W (2020). CircRNA inhibits DNA damage repair by interacting with host gene. Mol Cancer.

[39] Sang Y, Chen B, Song X, Li Y, Liang Y, Han D (2019). circRNA_0025202 regulates tamoxifen sensitivity and tumor progression via regulating the miR-182-5p/FOXO3a Axis in breast cancer. Mol Ther.

[40] Xu JZ, Shao CC, Wang XJ, Zhao X, Chen JQ, Ouyang YX (2019). circTADA2As suppress breast cancer progression and metastasis via targeting miR-203a-3p/SOCS3 axis. Cell Death Dis.

[41] Fan Y, Wang J, Jin W, Sun Y, Xu Y, Wang Y (2021). CircNR3C2 promotes HRD1-mediated tumor-suppressive effect via sponging miR-513a-3p in triple-negative breast cancer. Mol Cancer.

[42] Zheng X, Huang M, Xing L, Yang R, Wang X, Jiang R (2020). The circRNA circSEPT9 mediated by E2F1 and EIF4A3 facilitates the carcinogenesis and development of triple-negative breast cancer. Mol Cancer.

[43] Yin WB, Yan MG, Fang X, Guo JJ, Xiong W, Zhang RP (2018). Circulating circular RNA hsa_circ_0001785 acts as a diagnostic biomarker for breast cancer detection. Clin Chim Acta.

[44] He R, Liu P, Xie X, Zhou Y, Liao Q, Xiong W (2017). circGFRA1 and GFRA1 act as ceRNAs in triple negative breast cancer by regulating miR-34a. J Exp Clin Cancer Res.

[45] Wang X, Xing L, Yang R, Chen H, Wang M, Jiang R (2021). The circACTN4 interacts with FUBP1 to promote tumorigenesis and progression of breast cancer by regulating the expression of proto-oncogene MYC. Mol Cancer.

[46] Li J, Ma M, Yang X, Zhang M, Luo J, Zhou H (2020). Circular HER2 RNA positive triple negative breast cancer is sensitive to Pertuzumab. Mol Cancer.

[47] Chen B, Wei W, Huang X, Xie X, Kong Y, Dai D (2018). circEPSTI1 as a prognostic marker and mediator of triple-negative breast cancer progression. Theranostics.

[48] Hansen TB, Jensen TI, Clausen BH, Bramsen JB, Finsen B, Damgaard CK (2013). Natural RNA circles function as efficient microRNA sponges. Nature.

[49] Bartel DP (2018). Metazoan MicroRNAs. Cell.

[50] Zhang ZY, Gao XH, Ma MY, Zhao CL, Zhang YL, Guo SS (2020). CircRNA_101237 promotes NSCLC progression via the miRNA-490-3p/MAPK1 axis. Sci Rep.

[51] Wang L, Zhou Y, Jiang L, Lu L, Dai T, Li A (2021). CircWAC induces chemotherapeutic resistance in triple-negative breast cancer by targeting miR-142, upregulating WWP1 and activating the PI3K/AKT pathway. Mol Cancer.

[52] Li J, Gao X, Zhang Z, Lai Y, Lin X, Lin B (2021). CircCD44 plays oncogenic roles in triple-negative breast cancer by modulating the miR-502-5p/KRAS and IGF2BP2/Myc axes. Mol Cancer.

[53] Xu G, Ye D, Zhao Q, He R, Ma W, Li Y (2020). circNFIC suppresses breast cancer progression by sponging miR-658. J Cancer.

[54] Zhang XY, Mao L (2021). Circular RNA Circ_0000442 acts as a sponge of MiR-148b-3p to suppress breast cancer via PTEN/PI3K/Akt signaling pathway. Gene.

[55] Vera-Ramirez L, Vodnala SK, Nini R, Hunter KW, Green JE (2018). Autophagy promotes the survival of dormant breast cancer cells and metastatic tumour recurrence. Nat Commun.

[56] Chang CH, Bijian K, Wernic D, Su J, da Silva SD, Yu H (2019). A novel orally available seleno-purine molecule suppresses triple-negative breast cancer cell proliferation and progression to metastasis by inducing cytostatic autophagy. Autophagy.

[57] Liang G, Ling Y, Mehrpour M, Saw PE, Liu Z, Tan W (2020). Autophagy-associated circRNA circCDYL augments autophagy and promotes breast cancer progression. Mol Cancer.

[58] Dixon SJ, Stockwell BR (2014). The role of iron and reactive oxygen species in cell death. Nat Chem Biol.

[59] Liou GY, Storz P (2010). Reactive oxygen species in cancer. Free Radic Res.

[60] Hecht F, Pessoa CF, Gentile LB, Rosenthal D, Carvalho DP, Fortunato RS (2016). The role of oxidative stress on breast cancer development and therapy. Tumour Biol.

[61] Chen CY, Sarnow P (1995). Initiation of protein synthesis by the eukaryotic translational apparatus on circular RNAs. Science.

[62] Legnini I, Di Timoteo G, Rossi F, Morlando M, Briganti F, Sthandier O (2017). Circ-ZNF609 is a circular RNA that can be translated and functions in myogenesis. Mol Cell.

[63] Zhang M, Huang N, Yang X, Luo J, Yan S, Xiao F (2018). A novel protein encoded by the circular form of the SHPRH gene suppresses glioma tumorigenesis. Oncogene.

[64] Ma Y, Zheng L, Gao Y, Zhang W, Zhang Q, Xu Y (2021). A comprehensive overview of circRNAs: emerging biomarkers and potential therapeutics in gynecological cancers. Front Cell Dev Biol.

[65] Zhang Y, Jiang J, Zhang J, Shen H, Wang M, Guo Z (2021). CircDIDO1 inhibits gastric cancer progression by encoding a novel DIDO1-529aa protein and regulating PRDX2 protein stability. Mol Cancer.

[66] Conn VM, Hugouvieux V, Nayak A, Conos SA, Capovilla G, Cildir G (2017). A circRNA from SEPALLATA3 regulates splicing of its cognate mRNA through R-loop formation. Nat Plants.

[67] Ma N, Pan J, Wen Y, Wu Q, Yu B, Chen X (2021). circTulp4 functions in Alzheimer's disease pathogenesis by regulating its parental gene, Tulp4. Mol Ther.

[68] Zang J, Lu D, Xu A (2020). The interaction of circRNAs and RNA binding proteins: an important part of circRNA maintenance and function. J Neurosci Res.

[69] Janga SC, Mittal N (2011). Construction, structure and dynamics of post-transcriptional regulatory network directed by RNA-binding proteins. Adv Exp Med Biol.

[70] Abdelmohsen K, Panda AC, Munk R, Grammatikakis I, Dudekula DB, De S (2017). Identification of HuR target circular RNAs uncovers suppression of PABPN1 translation by CircPABPN1. RNA Biol.

[71] Xia P, Wang S, Ye B, Du Y, Li C, Xiong Z (2018). A circular RNA protects dormant hematopoietic stem cells from DNA sensor cGAS-mediated exhaustion. Immunity.

[72] Song X, Liang Y, Sang Y, Li Y, Zhang H, Chen B (2020). circHMCU promotes proliferation and metastasis of breast cancer by sponging the let-7 family. Mol Ther Nucleic Acids.

[73] Tang H, Huang X, Wang J, Yang L, Kong Y, Gao G (2019). circKIF4A acts as a prognostic factor and mediator to regulate the progression of triple-negative breast cancer. Mol Cancer.

[74] Li Z, Chen Z, Feng Y, Hu G, Jiang Y (2020). CircMMP11 acts as a ce-circRNA in breast cancer progression by regulating miR-1204. Am J Transl Res.

[75] Kong Y, Yang L, Wei W, Lyu N, Zou Y, Gao G (2019). CircPLK1 sponges miR-296-5p to facilitate triple-negative breast cancer progression. Epigenomics.

[76] Zhang X, Su X, Guo Z, Jiang X, Li X (2020). Circular RNA La-related RNA-binding protein 4 correlates with reduced tumor stage, as well as better prognosis, and promotes chemosensitivity to doxorubicin in breast cancer. J Clin Lab Anal.

[77] Yu XP, Liu CG, Qiu F, Xu YQ, Xing F, Yin JQ (2020). CircRNA_100395 protects breast carcinoma deterioration by targeting MAPK6. Eur Rev Med Pharmacol Sci.

[78] Yi Z, Li Y, Wu Y, Zeng B, Li H, Ren G (2020). Circular RNA 0001073 attenuates malignant biological behaviours in breast cancer cell and is delivered by nanoparticles to inhibit mice tumour growth. Onco Targets Ther.

[79] Lü L, Sun J, Shi P, Kong W, Xu K, He B (2017). Identification of circular RNAs as a promising new class of diagnostic biomarkers for human breast cancer. Oncotarget.

[80] Li Y, Li H (2020). Circular RNA VRK1 correlates with favourable prognosis, inhibits cell proliferation but promotes apoptosis in breast cancer. J Clin Lab Anal.

[81] Ye F, Gao G, Zou Y, Zheng S, Zhang L, Ou X (2019). circFBXW7 inhibits malignant progression by sponging miR-197-3p and encoding a 185-aa protein in triple-negative breast cancer. Mol Ther Nucleic Acids.

[82] Lin X, Chen W, Wei F, Xie X (2021). TV-circRGPD6 nanoparticle suppresses breast cancer stem cell-mediated metastasis via the miR-26b/YAF2 axis. Mol Ther.

[83] Chen N, Zhao G, Yan X, Lv Z, Yin H, Zhang S (2018). A novel FLI1 exonic circular RNA promotes metastasis in breast cancer by coordinately regulating TET1 and DNMT1. Genome Biol.

[84] Du WW, Yang W, Li X, Awan FM, Yang Z, Fang L (2018). A circular RNA circ-DNMT1 enhances breast cancer progression by activating autophagy. Oncogene.

[85] Du WW, Yang W, Li X, Fang L, Wu N, Li F (2020). The circular RNA circSKA3 binds integrin β1 to induce invadopodium formation enhancing breast cancer invasion. Mol Ther.

[86] He AT, Liu J, Li F, Yang BB (2021). Targeting circular RNAs as a therapeutic approach: current strategies and challenges. Signal Transduct Target Ther.

[87] Draz MS, Fang BA, Zhang P, Hu Z, Gu S, Weng KC (2014). Nanoparticle-mediated systemic delivery of siRNA for treatment of cancers and viral infections. Theranostics.

[88] Piwecka M, Glažar P, Hernandez-Miranda LR, Memczak S, Wolf SA, Rybak-Wolf A, et al. Loss of a mammalian circular RNA locus causes miRNA deregulation and affects brain function. Science. 2017:357. 10.1126/science.aam8526. 10.1126/science.aam8526 28798046

[89] Zhang Y, Nguyen TM, Zhang XO, Wang L, Phan T, Clohessy JG (2021). Optimized RNA-targeting CRISPR/Cas13d technology outperforms shRNA in identifying functional circRNAs. Genome Biol.

[90] Jiang Q, Liu C, Li CP, Xu SS, Yao MD, Ge HM (2020). Circular RNA-ZNF532 regulates diabetes-induced retinal pericyte degeneration and vascular dysfunction. J Clin Invest.

[91] Yang W, Du WW, Li X, Yee AJ, Yang BB (2016). Foxo3 activity promoted by non-coding effects of circular RNA and Foxo3 pseudogene in the inhibition of tumor growth and angiogenesis. Oncogene.

[92] Chen YG, Chen R, Ahmad S, Verma R, Kasturi SP, Amaya L (2019). N6-Methyladenosine modification controls circular RNA immunity. Mol Cell.

[93] Oliveira ACN, Fernandes J, Gonçalves A, Gomes AC, Oliveira M (2019). Lipid-based nanocarriers for siRNA delivery: challenges, strategies and the lessons learned from the DODAX: MO liposomal system. Curr Drug Targets.

[94] Wang X, Zhang H, Yang H, Bai M, Ning T, Deng T (2020). Exosome-delivered circRNA promotes glycolysis to induce chemoresistance through the miR-122-PKM2 axis in colorectal cancer. Mol Oncol.

[95] Salzman J, Gawad C, Wang PL, Lacayo N, Brown PO (2012). Circular RNAs are the predominant transcript isoform from hundreds of human genes in diverse cell types. PLoS One.

[96] Naeli P, Pourhanifeh MH, Karimzadeh MR, Shabaninejad Z, Movahedpour A, Tarrahimofrad H (2020). Circular RNAs and gastrointestinal cancers: epigenetic regulators with a prognostic and therapeutic role. Crit Rev Oncol Hematol.

